# Mental Health Trajectories Across the COVID‐19 Pandemic: A Cross‐Population Analysis of Harmonised Data

**DOI:** 10.1002/alz70860_102141

**Published:** 2025-12-23

**Authors:** James Lian, Sarah Bauermeister

**Affiliations:** ^1^ Dementias Platform UK, Oxford, Oxfordshire, United Kingdom; ^2^ University of Oxford, Oxford, Oxfordshire, United Kingdom; ^3^ Dementias Platform UK ‐ University of Oxford, Oxford, United Kingdom

## Abstract

**Background:**

This study leveraged harmonised longitudinal data to examine and compare mental health trajectories in older adults before, during, and after the COVID‐19 pandemic. The COVID‐19 pandemic has had profound and enduring effects on mental health worldwide. To understand the complexity of these impacts, the COVID Global Mental Health Consortium (CGMHC) aims to harmonise data across international cohorts, enabling comprehensive investigations. This study focuses on data from the English Longitudinal Study of Ageing (ELSA‐UK) and ELSA‐Brazil to explore cross‐population trajectories of mental health.

**Method:**

We developed harmonisation protocols to standardise data from the two cohorts, accounting for variations in study design, cultural context, and measurement tools. Multiple‐group confirmatory factor analysis (MG‐CFA) was conducted to establish measurement invariance of key mental health variables, ensuring comparability across the cohorts. Using the harmonised data, we applied latent growth curve modelling to investigate changes in depressive symptoms and anxiety levels, incorporating pre‐pandemic baseline data and longitudinal follow‐ups during and after the pandemic.

**Result:**

The harmonisation process successfully aligned data from ELSA‐UK and ELSA‐Brazil, with MC‐CFA confirming measurement invariance across mental health variables. Preliminary findings revealed notable differences in mental health trajectories between the cohorts, underscoring the impact of socio‐cultural and contextual factors.

**Conclusion:**

This study demonstrates the value of harmonising cross‐cultural data to address complex global health challenges. Integrating data from diverse populations provides nuanced insights into the mental health impacts of the COVID‐19 pandemic on older adults, offering evidence to inform targeted interventions and policy development.

